# Albany County Medical Society, Semi-Monthly Meeting

**Published:** 1874-12

**Authors:** F. C. Curtis


Adjourned.

———:o:———

ART. II.—*Albany County Medical Society. Semi-Monthly Meeting held Nov. 25th,* 1874. Reported by F. C. CURTIS, M. D.

The Society met at the usual time and place, Dr. JAS. S. BAILEY, President, in the chair.

The name of Dr. Thomas M. Trego, was proposed for membership, and referred to the Comitia Minora.

Dr. LEWIS BALCH reported a case of *Epulis of the Lower Jaw, (Malignant).*

The patient, a lad of 8 years, was admitted to St. Peter's Hospital, Oct. 18th. About a year previous, the right canine tooth was noticed to have grown inside of the incisor next to it. The latter was extracted and a "lump of flesh" grew in its place. This did not increase in size until about five months before admission. At that time the canine tooth was extracted and the growth incised, nothing but blood following the incision. It then grew rapidly and bled every night.

His family history is good. He has been in the habit of smoking cigar-stumps picked up in the street, and has also chewed tobacco.

Examination shows a dark colored, fungous growth in the right side of the mouth, part in front and part behind the teeth, having its base in the lower jaw. It pushed before it the incisor teeth, crowding them on to their neighbors, rendering the line of the teeth very uneven. It was decided to remove this, which was done, the growth simply being taken away, although the bone was largely implicated, on account of the amount of hemorrhage, leaving the removal of the latter to a second operation. The hemorrhage was controlled by pressue with picked lint. Fluid diet was ordered.

Eleven days later, the first operation being recovered from, the patient was again etherized and the affected bone removed. An incission three and a half inches long was made along the under edge of the jaw, from a point about an inch and a quarter to the left of the median line, to the right facial artery, the vessel not being severed. The flap thus made was turned up over the mouth, and the bony part of the tumor removed by bone forceps. The object in making the incission in the manner stated, was, if the disease was found to extend through the whole depth of the jaw, excision could be performed. Healthy bone was found, however, below the alveolar processes. Two ligatures were applied and the wound closed by three hare lip pins, with intermediate sutures of fine silk, a packing of picked lint were applied on the inside.

The pins were removed on the third day and the sutures on the fifth. The wound healed kindly by first intention, almost throughout. In two weeks the external wound was entirely healed. A few pouting granulations continued to spring up from the internal wound, which were touched with chloride of zinc. It healed by second intention. At first a lotion of nitric acid, a drachm to the pint of water, was used as a wash.

Drs. BENJAMIN and BECKETT each reported having met with a similar case. Dr. Benjamin supposed it to have originated in his case from an ulcerated tooth as an exciting cause.

Dr. O. H. E. CLARKE read a paper on *Strangulated Femoral Hernia*, and reported a case.

The patient, ten years ago, brought on a hernia by lifting. It was not noticed for two years, when it was increased by the same cause, and returned with difficulty. Again in January 1873, when she was seen for the first time by Dr. Clarke, she had a second attack of the same nature. It then appeared to be an entero-epiplocele. The tumor, the size of a fowl's egg was tense and painful, and in general condition the patient was much prostrated. A poultice containing two ounces of laudanum was applied, and one-third gr. morphia was given which procured an hour of sleep, after which the hernia was returned. In March 1874, the patient being sixty years of age, had strangulation take place. She had vomiting, pain, and was much prostrated. Taxis failing, as before opiates were given and a poultice applied. Sleep was not procured and taxis was again attempted. Among the expedients used were elevating the pelvis extremely while assistants held her by the right knee, the left limb (the side of the hernia) being bent to rather more than a right angle with the body and giving it a slight inclination to the other side; making her stand while reduction was attempted from behind, passing the arms around the abdomen; attempting reduction while she was placed on the hands and knees; attempting reduction after the operation of luke-warm water containing a little salt, thrown up the bowels. Nothing was effected beyond returning a little flatus. Morphia and a poultice containing laudanum were then again applied, (patient would not hear of cold applications) and she was left with pelvis elevated and the legs supported in a flexed position. After a time taxis was again attempted and persevered in with gentleness for an hour and a half, with intervals of rest.

The patient was then put under the influence of an anesthetic, and taxis again not proving successful, it was decided to operate. A transverse incision two inches in length and another perpendicular to it of three inches exposed the tumor. Instead of dividing the layers of fascia with a director and scalpel, a pair of small scissors with the point of one blade made blunt was used. They were believed to operate with more precission and expedition; minute portions can be cut, and in many respects they are safer. On opening the sac half a pint of serum ran out. The hernia was found to be omental, but a knuckle of intestine was believed to have been returned by taxis. The mass was about three inches long, very pale and firmly embraced by the falsiform border of the fascia lata. The index finger was inserted under this and it was cut in an upward direction freely. Attempts at reduction still not availing, the finger was introduced with some difficulty and the firm sharp edge of a small aperture was detected, the border of Gimbernat's ligament. This was cut in an upward and inward direction with a knife blunted to avoid injuring possibly the obturator artery. The omentum was then returned and carried a little to the right in the abdominal cavity. A few old adhesions to the sac had to be severed, and as they bled pretty freely were tied with fine silk dipped in a solution of carbolic acid before returning them. The same was thoroughly applied to the surfaces. Two string-like processes of omentum persisted in falling back, and were amputated.

The wound was closed with sutures dipped in carbolic acid, leaving a small opening for drainage, and the patient placed in bed, the foot of which was raised four inches, a small pillow being put under the thighs. Hot carbolized dressings were used. She did not rally very well, and on the third day the wound began to slough. This was excised freely, the sutures removed and a charcoal poultice made with carbolic acid lotion applied. Quinine and stimulants are also given. After a day or two flabby granulations sprang up. It was then packed with lint soaked in Edinburg red wash, consisting of zinci sulph. gr. 16, spts. rosemary and spts. lavandal. co. aa 2 drs. and carbolic acid lotion 8 ozs. A few days later an abscess formed in the back, which was opened and discharged very fetid matter. A large sinus formed in the lower part of the wound, and the patient was in a very reduced condition. Tonics, stimulants, and nourishment were pressed, and on the eighth day she began to improve. On the sixteenth day the wound was nearly healed. The recovery was complete, and the hernia appeared to be radically cured for a few months, but it has since come on again and has attained considerable size, as she will not wear a truss.

Points of special interest in this case are, the existence of enterocele with the epiplocele and its reduction under chloroform; the size of the tumor; the large amount of serum in the sac, there being generally but a few drams; and the return of this fluid under taxis into the abdomen, leading to delusive hopes that the hernia was being reduced, a point not mentioned by authors.

The paper closed with a discussion at length of the question as to how long operation should be deferred in strangulated hernia.

Numerous authorities were quoted, and the conclusion reached that we should operate the moment we are satisfied that taxis is not likely to succeed, and the patient's symptoms progressively increase in severity.

A vote of thanks to Dr. Clarke for his interesting paper, was carried.

Dr. MARCH thought the substitution of scissors for the scalpel and directer, in cutting down to a hernial sac, was a valuable suggestion.

Dr. BECKETT said, that early taxis was very important. He had found good results follow the use of a strong infusion of coffee, and believed that in one case where he had administered a quart of it, that it had been the means of making reduction by taxis possible.

Dr. HANNAN referred to a case under the care of a distinguished New York Surgeon, where operation was done three hours after the gut came down and the patient died three and a half hours afterwards. The length of time we should wait before operating cannot be fixed for every case.

Dr. WITBECK thought that the age of the patient influenced the result of the operation; the old are not so liable to inflammation as the young.

On motion adjourned.

